# Global Conformational Selection and Local Induced Fit for the Recognition between Intrinsic Disordered p53 and CBP

**DOI:** 10.1371/journal.pone.0059627

**Published:** 2013-03-26

**Authors:** Qingfen Yu, Wei Ye, Wei Wang, Hai-Feng Chen

**Affiliations:** 1 State Key Laboratory of Microbial metabolism, Department of Bioinformatics and Biostatistics, College of Life Sciences and Biotechnology, Shanghai Jiaotong University, Shanghai, China; 2 Shanghai Center for Bioinformation Technology, Shanghai, China; Uni. of South Florida, United States of America

## Abstract

The transactivation domain (TAD) of tumor suppressor p53 can bind with the nuclear coactivator binding domain (NCBD) of cyclic-AMP response element binding protein (CBP) and activate transcription. NMR experiments demonstrate that both apo-NCBD and TAD are intrinsic disordered and bound NCBD/TAD undergoes a transition to well folded. The recognition mechanism between intrinsic disordered proteins is still hotly debated. Molecular dynamics (MD) simulations in explicit solvent are used to study the recognition mechanism between intrinsic disordered TAD and NCBD. The average RMSD values between bound and corresponding apo states and Kolmogorov-Smirnov *P* test analysis indicate that TAD and NCBD may follow an induced fit mechanism. Quantitative analysis indicates there is also a global conformational selection. In summary, the recognition of TAD and NCBD might obey a local induced fit and global conformational selection. These conclusions are further supported by high-temperature unbinding kinetics and room temperature landscape analysis. These methods can be used to study the recognition mechanism of other intrinsic disordered proteins.

## Introduction

Cyclic-AMP response element binding protein (CBP) and its close relative protein p300 act as transcription coactivators that regulate transcription factors and chromatin *via* their intrinsic acetylase function [1], [2]. CBP and p300 comprise a number of modular binding domains, such as TAZ1, KIX, TAZ2, and the C-terminal nuclear receptor coactivator binding domain (NCBD) [3]. The NCBD subdomain can bind multiple proteins, including interferon regulatory factor IRF-3 [4], nuclear receptor coactivator ACTR [5] and tumor suppressor p53 [6]. The interactions between NCBD and the transactivation domain (TAD) of p53 are significant for p53 activating transcription upon binding to DNA as a tetramer [7]. The N-terminal TAD (residues 1–61) consists of two subdomains, termed AD1 (residues 1–42) and AD2 (residues 43–61) [8], [9]. Both AD1 and AD2 have contribution to bind NCBD [7].

**Figure 1:**
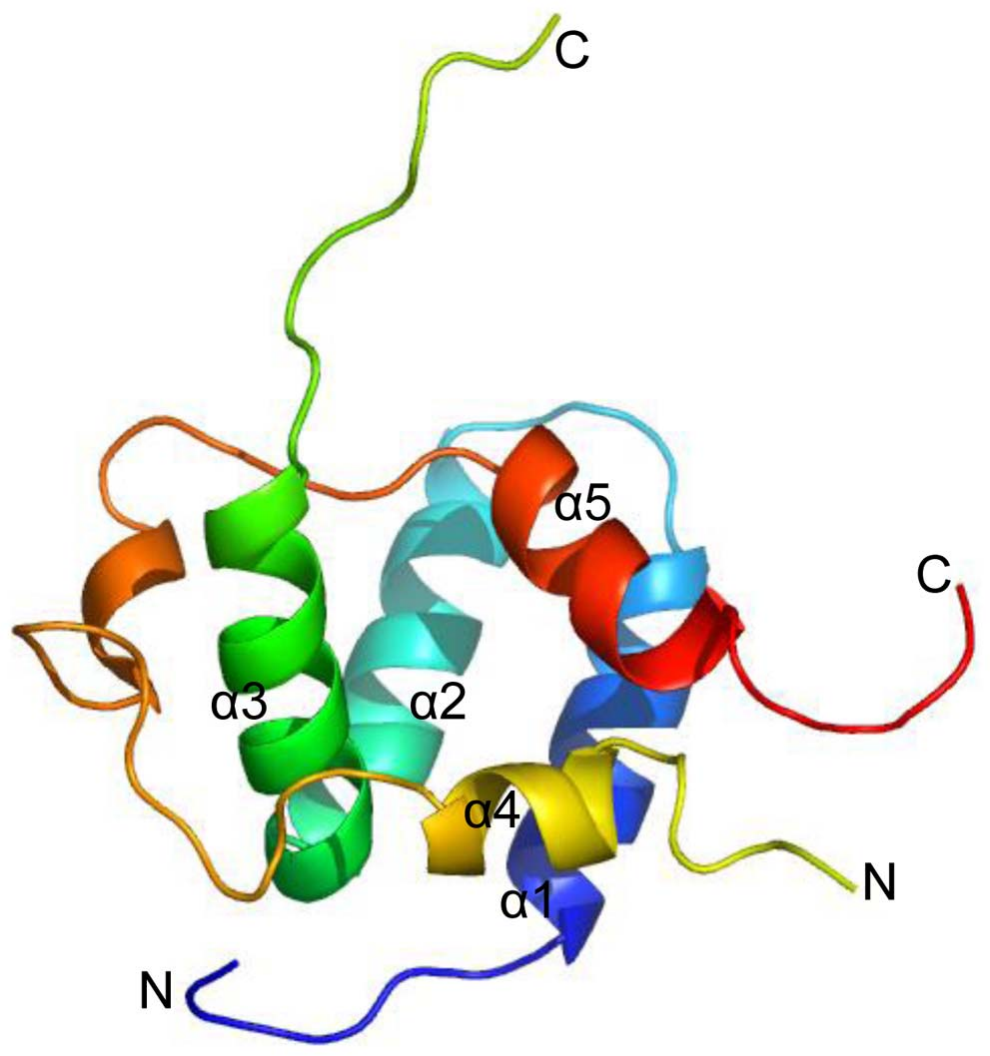
Ribbon representation of the NMR structure for TAD-NCBD complex (pdb code: 2L14). Helices α1, α2 and α3 of NCBD are colored with blue, cyan and green, respectively. Helices α4 and α5 of TAD are colored with yellow and red, respectively. N and C-terminal domains are labeled.large number of proteins (between 25% and 41%) are intrinsically disordered, however, these proteins also play important function in cell signaling and cancer upon binding with multiple interaction partners. [11] In this study, NMR experiments indicate that apo-TAD is intrinsic disordered protein and apo-NCBD is not entirely unstructured with a helical molten globule [3] [12]. Upon binding each other, both NCBD and TAD undergo a transition from disordered to well folded. [10] This suggests that both NCBD and TAD have significant conformational adjustment in complex. These experimental observations raise an interesting question if these intrinsic disordered NCBD and TAD obey an induced fit upon binding. To reveal this question, we utilize all atom molecular dynamics (MD) simulations in explicit solvent to analyze the coupling between binding and folding in the NCBD-TAD complex. [13].

The NMR structure of NCBD and TAD complex was released in 2010 (pdb code: 2L14) [10]. The complex has five α-helices: α1, α2, α3, α4, and α5. NCBD consists of helix α1 from Ser8 to Leu17, helix α2 from Pro23 to Lys34, and helix α3 from Pro37 to Arg47. TAD includes helix α4 within AD1 from Phe66 to Leu72, and helix α5 within AD2 from Pro94 to Trp100. The residues between helices α1 and α2 form an unstructured long loop from Phe74 to Ala86. The three helices α1, α2 and α3 form a broad hydrophobic cleft for TAD binding. The residues from Met87 to Leu92 of p53 TAD tend to form a distorted helix upon binding to NCBD. The structure of complex is shown in Figure 1.

A large number of proteins (between 25% and 41%) are intrinsically disordered, however, these proteins also play important function in cell signaling and cancer upon binding with multiple interaction partners. [11] In this study, NMR experiments indicate that apo-TAD is intrinsic disordered protein and apo-NCBD is not entirely unstructured with a helical molten globule [3] [12]. Upon binding each other, both NCBD and TAD undergo a transition from disordered to well folded. [10] This suggests that both NCBD and TAD have significant conformational adjustment in complex. These experimental observations raise an interesting question if these intrinsic disordered NCBD and TAD obey an induced fit upon binding. To reveal this question, we utilize all atom molecular dynamics (MD) simulations in explicit solvent to analyze the coupling between binding and folding in the NCBD-TAD complex [13].

However, so far the folding time scales of all atomic MD simulations are restricted to microsecond magnitude at room temperature (298K), which is significant shorter than the folding half times of most proteins [14], [15]. In order to reveal the conformational changes within reasonable time, all MD simulations in explicit solvent at high temperature have been widely used to monitor the unfolding pathways of proteins. The unfolding timescales could be nanosecond at 498K [14], [16]. Moreover, according to the principle of microscopic reversibility, experiments have demonstrated that the transition state for folding and unfolding is supposed to be same [14]. Therefore, MD simulations high temperatures are used in this study. Although it is impossible to accumulate long enough trajectories at room temperature to draw any meaningful conclusions, multiple trajectories of room temperature simulation are also used in this research to compare with experimental observations and other simulations.

## Materials and Methods

### 1. Molecular Dynamic Simulations

The atomic coordinates of NCBD and TAD were obtained from NMR structure (pdb code: 2L14) [10]. Point mutants of L10A/L13A, L69Q/W70S, W100Q/F101S and L69Q/W70S/W100Q/F101S were modeled with SCWRL3. [17] All hydrogen atoms were added using the LEAP module of AMBER 11 [18]. Counter-ions were used to maintain system neutrality. All systems were solvated in a truncated octahedron box of TIP3P waters with a buffer of 10 Å [19]. Particle Mesh Ewald (PME) [20] was applied to handle long-range electrostatic interactions with default setting in AMBER11 [18]. The parm99 force filed was used to compute the interactions within protein [21]. The SHAKE algorithm [22] was employed to constrain bonds including hydrogen atoms. All solvated systems were first minimized by 1000-step steepest descent to remove any structural clash, followed by 20 ps heating up and brief equilibration in the NPT ensembles at 298K. The time step was 2 fs with a friction constant of 1 ps^−1^ using in Langevin dynamics. To study the folded state of each solvated system, ten independent trajectories of 10.0 ns each in the NPT ensemble at 298K were simulated with PMEMD of AMBER11. Then ten independent unfolding trajectories of 10 ns each were performed to investigate unfolding pathways for each solvated system in the NVT ensemble. Four mutant systems were simulated for five trajectories of 10.0 ns each at 298K. A total of 800 ns trajectories were collected for the wild type and mutant at 298K and 498K. It took about 55,000 CPU hours in the in-house Xeon (3.0 GHz) cluster.

Native contacts of the bound and apo states for NCBD and TAD were monitored to detect the beginning of the unfolded state. It was found that 8 ns were sufficient to reach the equilibrium state for both apo and bound states at 498K. Therefore, the first 8 ns (a total of 80.0 ns for each system) were used to monitor the unbinding kinetics. The remaining 2 ns (a total of 20.0 ns for each system) were used to study the unfolded equilibrium state.

### 2. Transition State Analysis

The Cα root mean square deviations (RMSD) and conformational cluster at 498K were used to determine transition state [23]. The RMSD between any two conformers along the 498K trajectory was defined by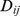. Suppose 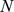
structures were extracted from a trajectory. These structures were mapped as 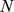 points 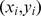 into two dimensions. The Euclidean distance of any two points was calculated with 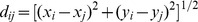. Suppose the 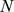 structures and the 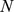 points in two-dimension are linear mapping, the error 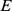 between them can be defined with equation (1). Nonlinear mapping algorithm (NLM) [24] was applied to optimize the error *E* between 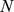 points and 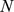 structures.
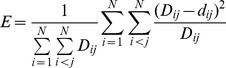
(1)


### 3. Data Analysis

The tertiary assignments were performed with in-house software [25], [26], [27], [28], [29], [30], [31]. The side chains of two residues that were not adjacent were supposed to be in contact when the distance was less than 6.5 Å. Electrostatic interactions are assigned when the distance between the center mass of positive charge and negative charge residues for NCBD and TAD is less than 11 Å [32]. The fraction of native tertiary contact (Qf) and the fraction of native binding contact (Qb) are used to reveal the unbinding kinetics. All fitted kinetic curves are handled with Origin 8.5.

According to the definition of protein Φ-values, the Φ-values were calculated with a similar method used in the previous works [33], [34], [35]

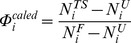
(2)where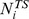 represents the number of native contacts of residue *i* at the transition state, 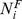 and 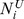
represents the number of native contacts of residue *i* at the folded and unfolded state, respectively. Contacts were assigned if the side chain heavy atoms of two residues that are nonadjacent were less than 6.5 Å.

### 4. Conformational Selection and Induced Fit Mechanism

For each simulated bound conformation in 298K trajectory, the apo conformation with the minimum RMSD was selected as the global most similar structure [36]. 10 pairs of bound conformations and apo conformations were used to evaluate the average RMSD as a function of distance from the centroid of binding partner.

To obtain the relationship between structural deviations and the distance from the centroid of binding partner, all atoms are assigned to different groups range from 0 to 50 Å at intervals of 0.5 Å [36] according to the distance from the centroid. The bound and apo conformations were superposed by all Cα atoms. The centroid of binding partner was defined as the center of mass in bound partner conformation.

For each pair of bound and apo conformation, the RMSD deviations were used to test the *Ρ* value. The two-sample Kolmogorov-Smirnov (KS) [37] test was used to reveal the distribution of RMSD values for bound conformation and apo conformation in each distance group. Both the median *P* value and the conformation fraction with *P*<0.1 were calculated for total 100 pairs of conformations in each distance group. Because the distributions of magnitudes of structural deviations do not fit any known distribution, therefore, the nonparametric KS test [38] was used in our study.

Histograms of average RMSD values between bound conformation and apo conformations were quantified as the magnitude of conformational selection and induced fit for total 100 pairs of conformations. The probabilistic weighting differences between distributions of conformational selection and induced fit [36] were used to determine the relative magnitude
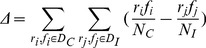
(3)where 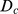
and 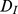
represent the distributions of deviation values for conformational selection and induced fit, respectively, 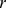
represent the RMSD values, 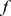
represent the frequencies of RMSD values, 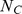 and 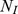 represent the number of points in distributions of RMSD values for conformational selection and induced fit, respectively.

## Results

### 1. Binding Mode between Intrinsic Disordered NCBD and TAD

**Figure 2:**
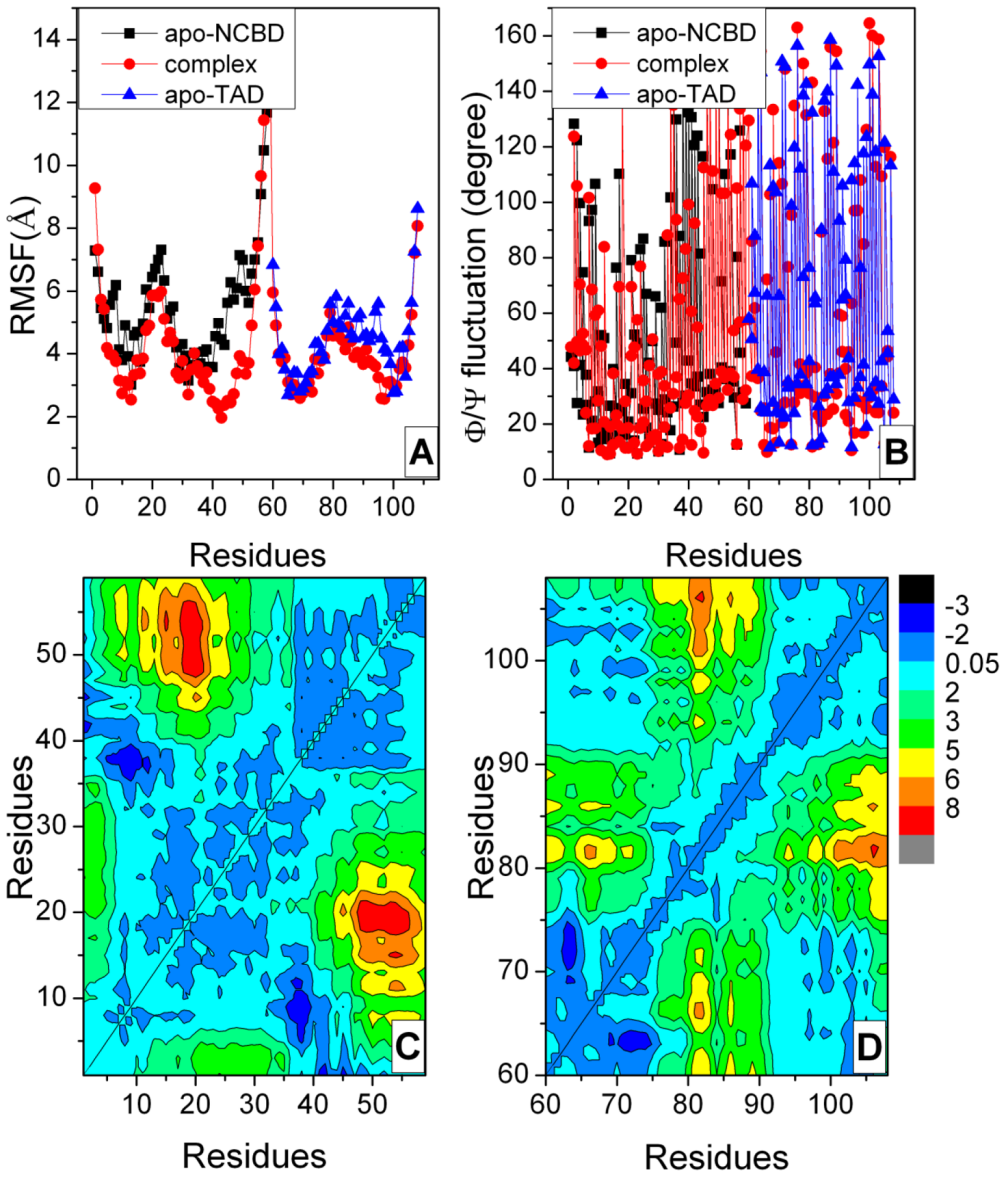
Variations and landscapes of distance difference for apo-NCBD, apo-TAD and complex. A: Cα atomic fluctuation for NCBD and TAD. B: Φ/Ψ variation for NCBD and TAD. C: landscapes of distance difference for NCBD. D: landscapes of distance difference for TAD.

A limited number of trajectories for MD simulations (5–10) are sufficient to capture the average properties of proteins [39]. To study the recognition for intrinsic disordered NCBD and TAD, 10 independent trajectories of 10.0 ns each for apo-NCBD, apo-TAD, and their complex were simulated at room temperature (298K), respectively. Cα and Φ/ψ fluctuations for apo and bound states are illustrated in Figure 2. The Cα variations of bound NCBD are significant smaller than those of apo-NCBD, especially in the region of C-terminal (residues Ile42 to Gly58) at the TAD binding site. This indicates that bound NCBD becomes more stable upon TAD binding. The Cα variations of bound TAD are also smaller than those of apo-TAD, especially in helix α5 (residue Pro94 to Trp100) within AD2 subdomain. It suggests that helix α5 becomes less flexible and more rigid upon NCBD binding. The Φ/ψ variation of bound NCBD is similar to that of apo-NCBD. This suggests that the secondary structure has not significant change upon TAD-binding. The Φ/ψ variation of bound TAD is similar to that of apo-TAD. These results are in good agreement with NMR experiments [10].

**Figure 3:**
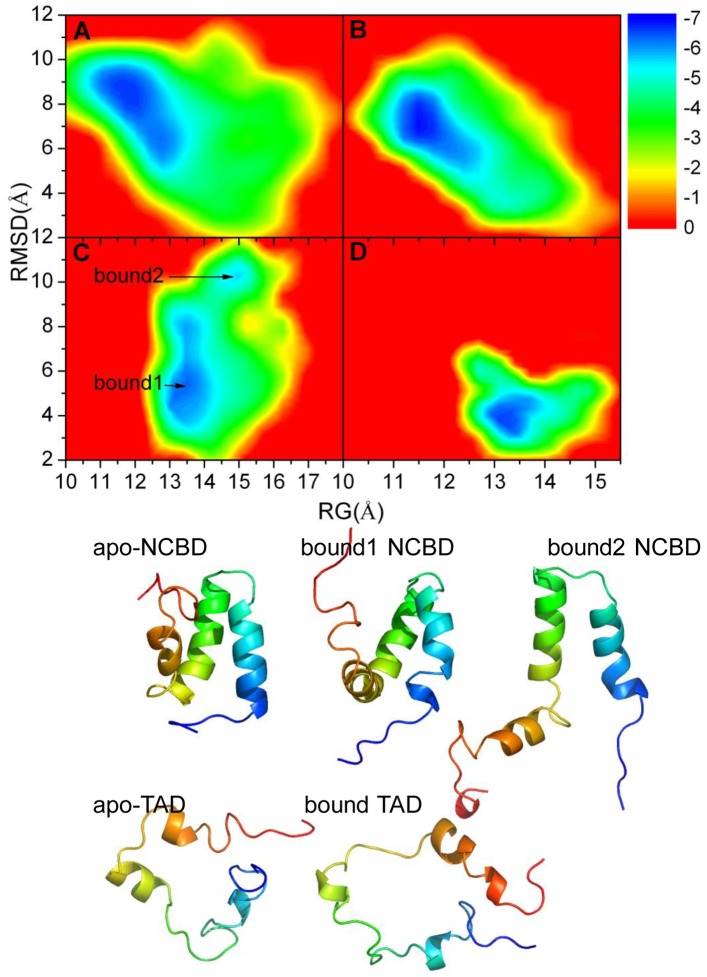
Free energy landscapes with respect to Rg and RMSD for apo and bound states of NCBD and TAD. A: apo-NCBD. B: apo-TAD. C: bound NCBD. D: bound TAD.

To clearly illustrate the conformational difference, the landscapes of distance difference between the average pairwise intra-molecular distance of bound states and corresponding average pairwise intra-molecular distance of apo states for NCBD and TAD are shown in Figure 2C–2D. The landscapes can reflect the relative conformational change of NCBD and TAD backbone. The deep red areas show that the distance differences between residues 5–30 and 40–59 for NCBD are positive values. This indicates that both helices α1 and α2 are stretched away from helix α3. The deep blue areas represent that the distance differences are negative values. The most regions of distance differences between bound and apo-NCBD residues are negative, indicating that the NCBD backbone become more rigid and structured upon p53 TAD binding. The landscape of distance differences between bound and apo-TAD residues is also shown in Figure 3. It is found that most distance differences are positive, especially the region of residues Glu75 to Leu90. This reflects that bound TAD is extended upon NCBD binding because the residues from Pro74 to Ala86 form a long distorted loop around NCBD helix α3 [10].

To explore the conformational difference between bound and apo states for NCBD and TAD, the energy landscapes with the reaction coordinates of RMSD and the radius of gyration (Rg) were shown in Figure 3. From this figure, we can find that apo-NCBD has the propensity of extension with the maximum Rg of 18 Å and RMSD of 12 Å for apo state (16.5 Å for bound NCBD). The distribution of conformer for apo-TAD is also looser than that of bound TAD. There are two basins of energy-minimum for bound NCBD, one basin (Bound1) with Rg values between 13 Å and 14 Å and Cα RMSD ranking between 4 Å and 8 Å, the other (Bound2) with Rg between 14.5 Å and 15 Å and Cα atom RMSD between 10 Å and 11 Å. There is just one energy-minimum basin for apo NCBD with Rg between 11 Å and 13.5 Å and Cα atom RMSD between 5 Å and 10 Å. For TAD, there is one energy-minimum basin for apo TAD with Rg between 11 Å and 13 Å and Cα atom RMSD between 5 Å and 8 Å, and one basin for bound TAD with Rg between 13 Å and 14 Å and Cα atom RMSD between 3 Å and 5 Å. The average structures corresponding to energy-minimum for NCBD and TAD are also shown in Figure 3. The helical content of apo NCBD is about 44%, which is lower than that of bound1 (47%) or bound2(54%). As for the conformers of two basins of bound1 and bound2 for bound NCBD, helix α3 of bound1 separates from helices α1 and α2. The helical content of apo TAD (28%) is similar to that of bound TAD (27%).

**Figure 4:**
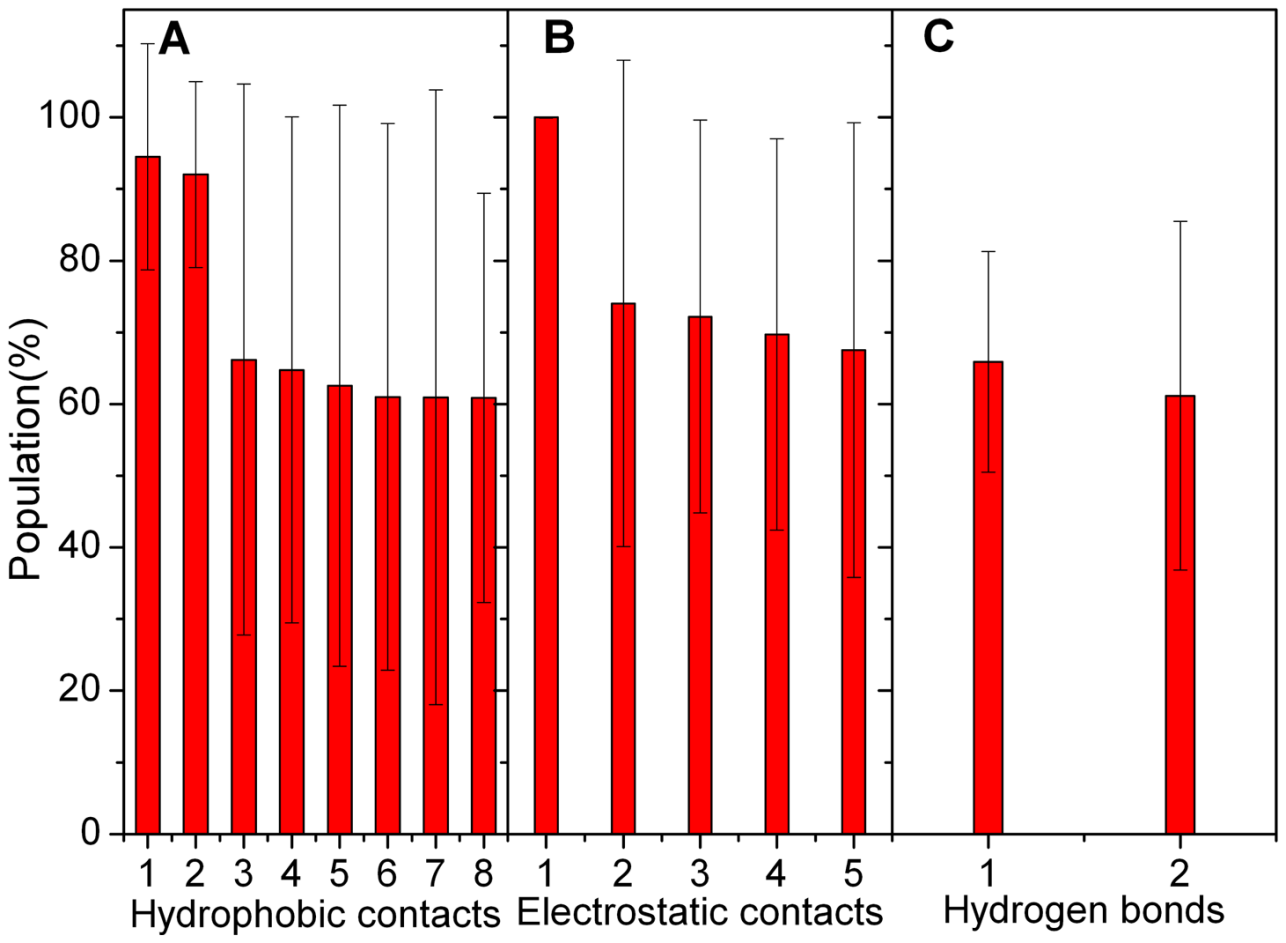
Interactions between NCBD and TAD. A: Hydrophobic contact. 1 for Ala42/Leu73; 2 for Ile44/Met87; 3 for Ala42/Pro74; 4 for Leu17/Phe101; 5 for Met40/Met87; 6 for Leu17/Ile97; 7 for Leu14/Phe101; and 8 for Phe43/Trp100. B: Electrostatic interaction. 1 for Arg47/Asp96; 2 for Lys45/Glu75; 3 for Lys50/Asp96; 4 for Arg3/Glu75; and 5 for Lys18/Asp104. C: Hydrogen bond. 1 for OD1(Asp96)/NH2(Arg47); and 2 for OD2(Asp96)/NH2(Arg47).

To study the driving force for these conformational adjustments, the electrostatic, hydrophobic, and hydrogen-binding interactions between NCBD and TAD were analyzed. Figure 4A illustrates the stable hydrophobic contacts in ten independent simulations. Eight stable hydrophobic contacts were found with population higher than 60%, such as Ala42/Leu73, Ile44/Met87, Phe43/Trp100, Ala42/Pro74, Leu17/Phe101, Met40/Met87, Leu17/Ile97, and Leu14/Phe101. It was found that the hydrophobic residues of Met87, Ile97, Trp100 and Phe101 are located at helix α5. This suggests that AD2 subdomain plays a key role in hydrophobic interactions between NCBD and TAD, consistent with the experimental observation [7], [10]. The electrostatic interactions are illustrated in Figure 4B. Five stable electrostatic interactions are found between NCBD and TAD. The positively charged and negatively charged residues are focused on Arg47/Asp96, Lys45/Glu75, Lys50/Asp96, Arg3/Glu75 and Lys18/Asp104, indicating that electrostatic contacts also have contributions to the stability of complex. Besides the hydrophobic and electrostatic interactions, there are also two stable hydrogen bonds and shown in Figure 4C. They are focused on Arg47 and Asp96. In summary, these interactions are located at helices of NCBD and TAD. This suggests that helical regions are critical for the binding of NCBD and TAD. The contribution of binding free energy between NCBD and TAD for hydrophobic residues is about 80% with the MMPBSA method (shown in Figure S1A). Therefore, hydrophobic contacts play key roles in the recognition between intrinsic disordered NCBD and TAD. This is in accord with most protein-protein interactions conclusion that the interface is predominant hydrophobicity [40].

### 2. Binding Kinetics

**Figure 5:**
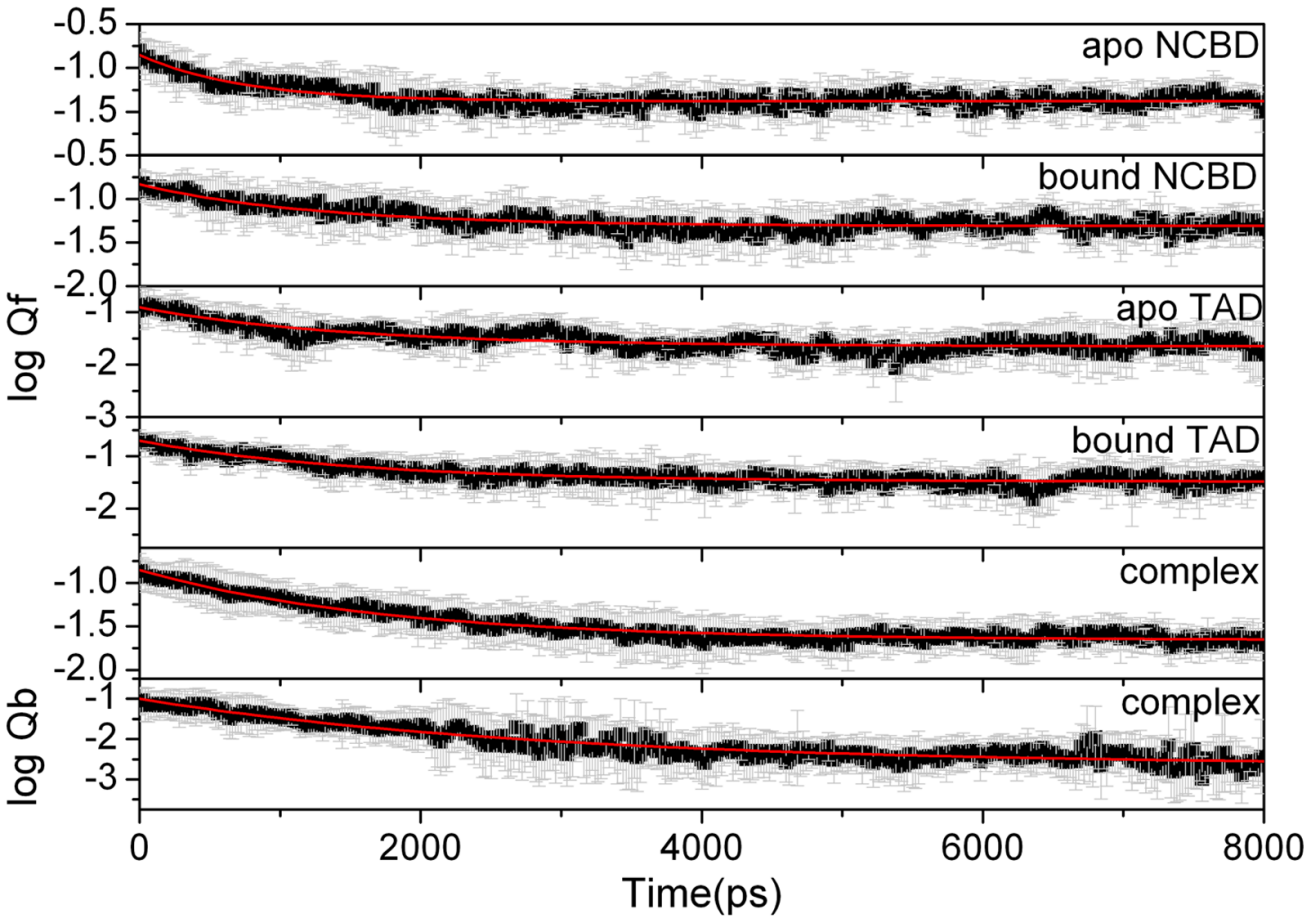
Kinetics fitting of Qf and Qb for apo and bound states for NCBD and TAD.

The fraction of native tertiary contact (Qf) and the fraction of native binding contact (Qb) in log scale are applied to reveal unfolding and unbinding kinetics. Time evolutions of logQb, logQf for apo and bound of NCBD and TAD, and logQf for the complex are shown in Figure 5. This figure suggests that the tertiary unfolding and unbinding can be fitted well by a single exponential function, indicating first order kinetics in the NVT ensemble at high temperature (498K). The fitted kinetics data were listed in Table 1. The kinetics analysis shows that the unbinding half-time is 2.918±0.028 ns, the tertiary unfolding half-time is 1.260±0.016 ns for bound NCBD, 1.732±0.010 ns for the complex, and 1.539±0.014 ns for bound TAD. This indicates that the tertiary unfolding of NCBD and TAD is slight faster than the unbinding between NCBD and TAD, respectively. The half-time of tertiary unfolding for apo-NCBD and apo-TAD was 0.724±0.010 ns and 1.473±0. 020 ns. This suggests that the tertiary unfolding of bound NCBD and TAD is slightly postponed upon the binding of each other.

### 3. Transition State Analysis

**Figure 6:**
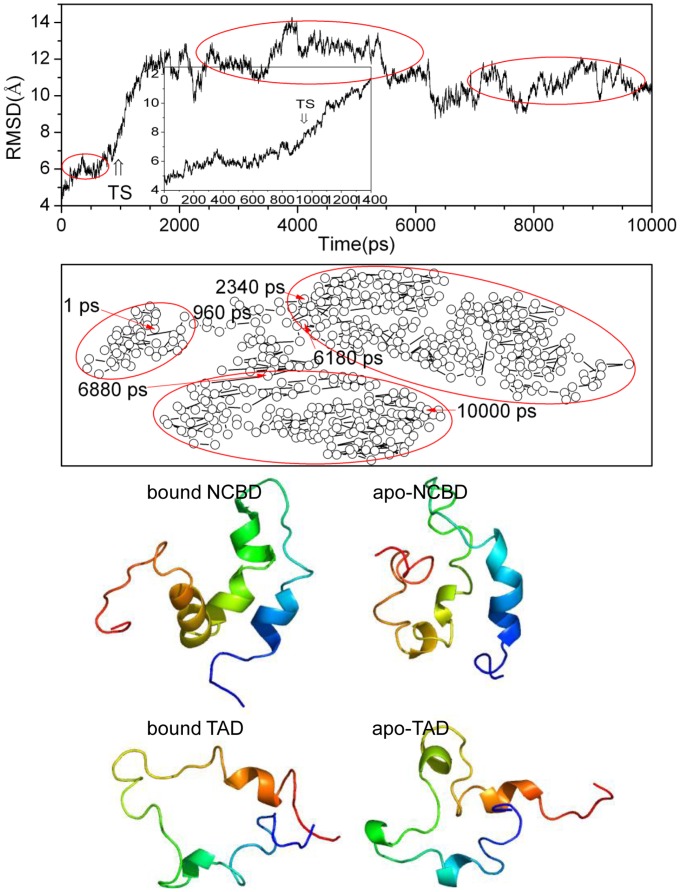
Identification of transition state. A: Cα RMSD of complex at 498K, the first 1400 ps in 498K trajectory are inset, the time point of transition state is labeled. B: two-dimension projection with root mean square deviation (RMSD), the time span of each cluster is labeled. C: average structure of transition state for bound and apo states of NCBD and TAD.

Based on the unfolding kinetics, the complex unfolds via a two-state process [41]. Therefore, a transition state corresponds to the energy-maximum along the reaction coordinate at high-temperature. Experiments have supported that the transition state gets close to the native state with kinetically and thermodynamically distorted [42], [43], [44]. Therefore, the Cα root mean square deviations (RMSD) between the high-temperature simulated complex and the NMR structure were first analyzed and shown in Figure 6. There are three plateaus in this figure. The first plateau between 500 and 800 ps represents a distorted structure from native state. The second plateau between 2000 and 6000 ps is a state with the structure sharply unfolding. The last plateau from 7000 to 10,000 ps corresponds a unfolded state. Between the first and the second plateau, the RMSDs have a significant improvement with 4 Å deviations corresponding to the loss of native tertiary contacts and the broken of hydrophobic core.

According to the analysis of RMSD, the structures corresponding to the energy-maximum comprise the transition sate. It is found that transition state occurs early to form a distorted structure of the native state. [45] The RMSD analysis is shown that the structures near 960 ps significantly change after temperature equilibration. This indicates that the structures around 960 ps might comprise the transition state ensemble.

In order to confirm the transition state, the conformer cluster [23] was analyzed to identify different states along trajectory at high temperature. Figure 6 illustrates the cluster analysis for the complex. Two dimensional projection of RMSD [24], [45] approximates the deviations of conformational space along the whole trajectory. All points are sequentially connected at intervals of 20 ps to form three distinct clusters. The first cluster ranges between 1 and 960 ps, the second one between 2340 and 6180 ps, and the last one between 6880 and 10,000 ps. These time spans of clusters are consistent with those of plateau regions. The first cluster includes the initial and the rapid structural deviations. This suggests that weak activation energy is sufficient for the initial structure rearrangements. Therefore, the activation free energy for the structural changes around 960 ps is the major barrier. After transformation from transition state, the structures enter into the second cluster. The last cluster is comprised of the structures that have lost most native tertiary contacts and represent the unfolded state. Based on the RMSD and cluster analysis, the transition state is composed of the structures around 960 ps.

The same procedures were applied to both apo-NCBD and apo-TAD, and yielded their transition states. Figure 6 also illustrates the average structures of transition states for apo-NCBD and bound NCBD. It is found that the transition state structure of bound NCBD is more native-like than that of apo-NCBD. In fact, the native hydrophobic contacts within bound NCBD are 32.76% and apo-NCBD with 27.59%. The average structures of transition states for both apo-TAD and bound TAD are also shown in Figure 6. The long loop between helices α4 and α5 is extended in bound TAD, mainly because the helices α4 and α5 surround the broad hydrophobic cleft formed by three helices of NCBD. Similar to NCBD, bound and apo-TAD are also denature-like. This is consistent with the NMR experiments [10].

### 4. Ф-value Prediction

**Figure 7:**
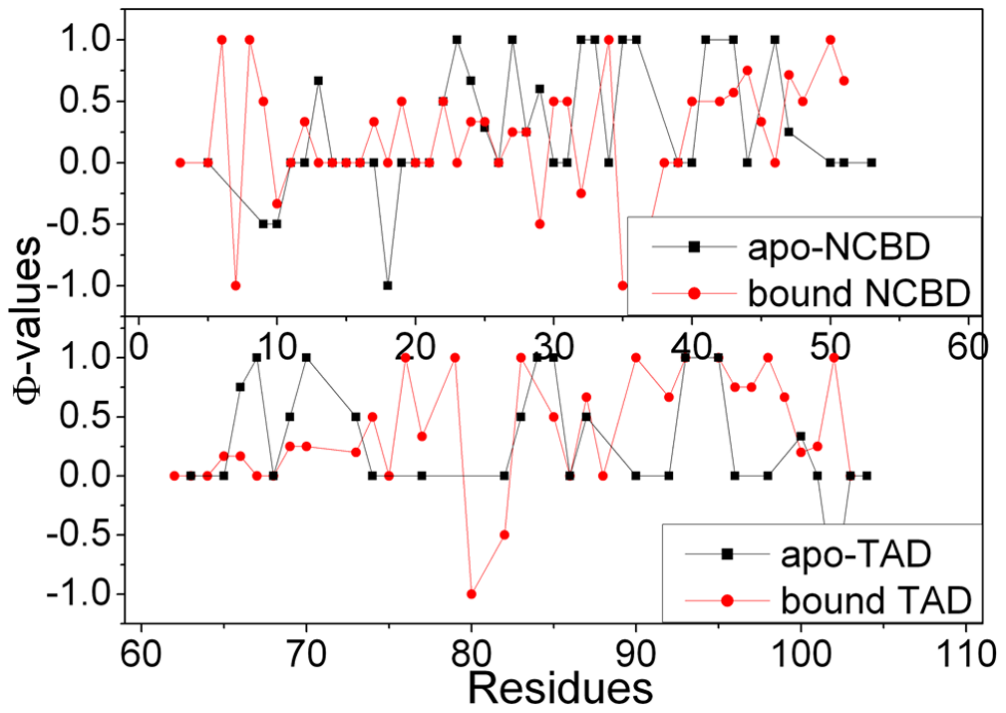
Predicted Ф-values for apo and bound states for NCBD and TAD.

Ф values have been widely used by theoretical and experimental works to identify the key residues for protein folding [46], [47], [48]. The structures of transition state ensemble are used to predict the Ф values and shown in Figure 7. This figure illustrates that the Ф values of helix α1, α2, α3, α4, and α5 between bound and apo states have not significant different. To our surprise, the main difference is focused on the loop regions (residues Pro74 to Ala86) between helices α4 and α5 and relative larger Ф values in bound TAD. In addition, residues Met87, Asp88, Leu90 and Leu92 have larger Ф values. This is consistent with the experimental observation that these residues tend to form a distorted helix for the complex [10]. All predicted Ф values can be confirmed by experiments.

## Discussion

### 1. Comparison with Experiment

The NMR structure of NCBD/TAD complex indicates that the hydrophobic residues Leu10, Leu13, Leu14, Leu17, Val29, Leu30, Leu33, Leu39, Met40, Ala42, Phe43, and Ile44 in helices Cα1, Cα2 and Cα3, provide contacts with hydrophobic residues Phe66, Trp70, Leu73, Met87, Leu90, Met91, Leu92, Ile97, Trp100, Phe101 in both AD1 and AD2 subdomains [10]. These contacts are critical for the stability of complex. Our room temperature simulations found 8 stable hydrophobic contacts with populations higher than 60% and shown in Figure 5A. These hydrophobic residues can stabilize the complex and consistent with the experiment. Our Φ-value analysis also confirms these key residues.

Experimental point mutation shows that L10A/L13A, L69Q/W70S, W100Q/F101S, L69Q/W70S/W100Q/F101S have significant effect on the recognition of intrinsic disordered TAD and NCBD. [49], [50] Indeed our predicted binding free energy lost is about 7 kcal/mol with −91.262±6.337 kcal/mol for WT and −84.074±12.911 kcal/mol for L10A/L13A mutant using the MMPBSA method. [51] The binding free energy is listed in Table 2. The binding free energy is also lost 19 kcal/mol with −72.743±16.00kcal/mol for L69Q/W70S, 9 kcal/mol with −82.100±6.006 kcal/mol for W100Q/F101S, 13 kcal/mol with −68.627±13.945 kcal/mol for L69Q/W70S/W100Q/F101S, respectively. This suggests that L10/L13, W100/F101, L69/W70/W100/F101 are key residues for the stability of NCBD-TAD and consistent with the previous mutant experiment. [49], [50] The results of binding free energy with Poisson-Boltzmann method are similar to those of Generalized Bond method.

### 2. Recognition Mechanism

Conformational selection and induced fit are two widely used models to interpret the recognition between intrinsic disordered proteins [52]. According to the conformational selection paradigm, various conformational ensembles explore the free energy landscapes corresponding to diverse stable unbound states in equilibrium. During the binding process, the favorable conformation compatible with binding selectively stabilize, and the populations of conformational ensembles shift towards stabilizing state [53], [54], [55], [56]. However, the induced fit scenario proposes that the favorable conformation results from significant changes of unbound ensembles upon allosteric binding [57], [58], [59], [60]. It is worthy to point out that conformational selection and induced fit models cannot be distinguished absolutely [61]. Indeed, some systems involve kinetic elements of both mechanisms [62], [63].

**Figure 8:**
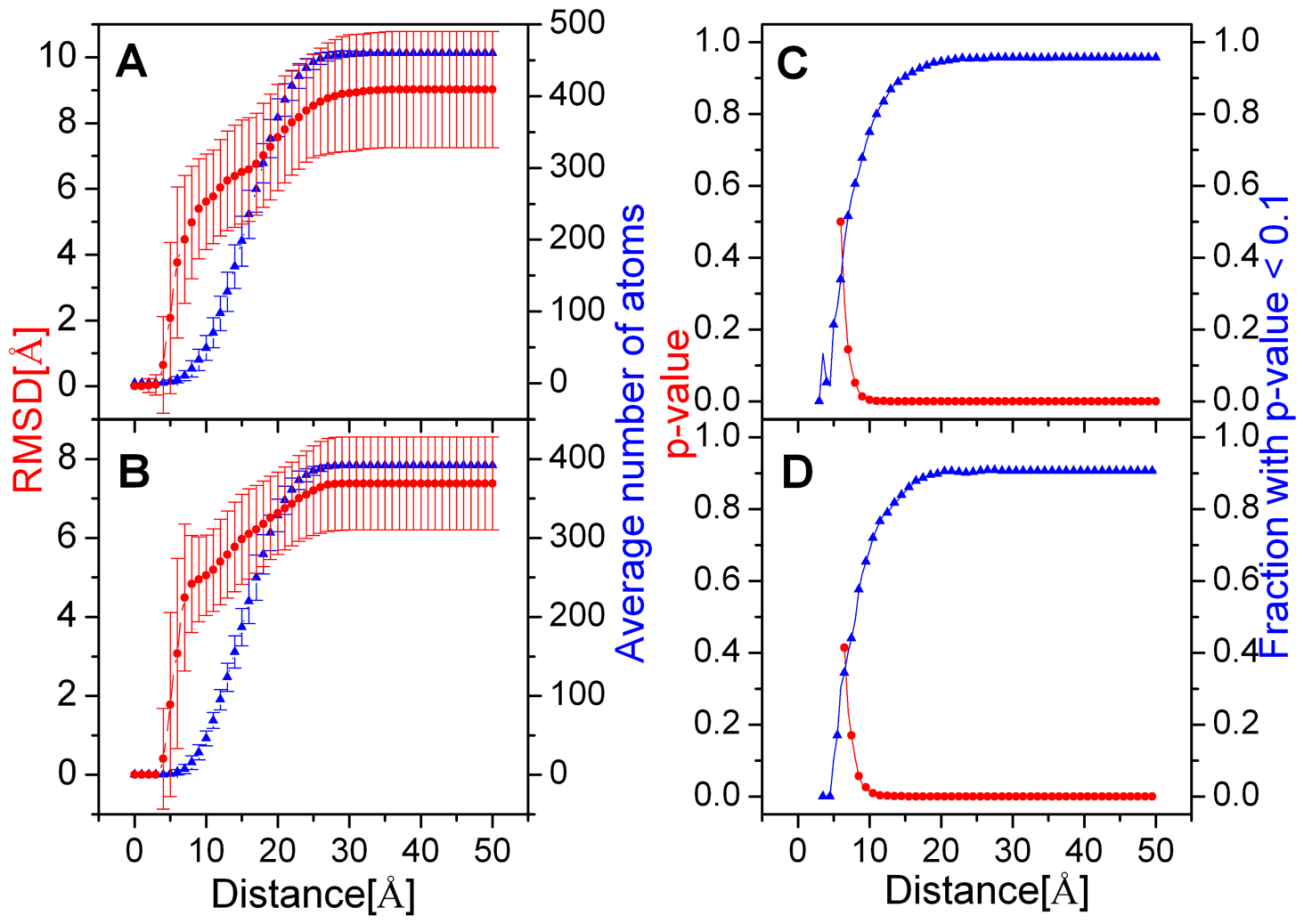
Local conformational RMSD differences between bound and apo conformations as a function of distance from the centroid of binding partner and statistical significance of conformational selection in NCBD and TAD binding. Average local RMSD for 10 pairs of bound conformations and the most similar apo conformation and for 90 pairs of bound NCBD and the other apo conformations, as a function of distance from the centroid of binding partner. A: NCBD. B: TAD. C: NCBD. D: TAD.

For this system, we analyze the structural deviations of NCBD and TAD upon binding. The possible magnitudes of conformational selection and induced fit [36] are calculated to reveal the recognition mechanism. To explore the recognition mechanism, the average RMSD deviations of bound conformation and apo conformations are analyzed as a function of distance from the centroid of binding partner and shown in Figure 8. This figure illustrates that the RMSD variation gradually increases until to the global level. This suggests that there is an induced fit far away for the binding site. Similar results are found for TAD (shown in Figure 8B).

To address the statistical significance for differences of deviations between these two systems, two sample Kolmogorov-Smirnov test [37] is used to calculate the *P* value for each distance group. Figure 8C illustrates the median of *P* values and the fraction with *P*<0.1 for all 100 pairs of NCBD conformations in each distance group. It is found that the median *P* values are typically smaller than 0.1 in most distance group, especially in some larger distance group with median *P* values approximates 0. The conformations with *P*<0.1 exceed 50% in most distance groups. This suggests that the bound NCBD is significant different from the apo conformation far away from the binding site and the differences are statistically significant. Similar results are found for 100 pairs of p53 TAD conformations. Based on the RMSD and p-value analysis, the recognition between intrinsic disordered NCBD and TAD might obey an induced fit.

Average RMSD and KS test analysis suggest the possibility of induced fit in the recognition between intrinsic disordered NCBD and TAD. Next natural question to ask is if there is any global conformational selection and the relative magnitudes of induced fit and conformational selection in the NCBD-TAD recognition. The histograms of conformational frequency for induced fit and conformational selection are used to evaluate the relative magnitudes. Parameter Δ represents the probability-weighted difference between conformational selection and induced fit at global and local regions and is shown in Figure S1B. This figure suggests that the magnitude of conformational selection is quantitative larger than that of induced fit at global level, and smaller than that of induced fit at local level. This indicates that NCBD might adopt global conformational selection and local induced fit upon TAD binding. Furthermore, the global magnitude of conformational selections is also larger than that of induced fit and local magnitude of conformational selection is also smaller than that of induced fit for TAD. It reveals that TAD might also obey global conformational selection and local induced fit mechanism. These findings are consistent with the previous works that the intrinsic disorder protein [64] obeys conformational selection and induced fit mechanism [11], [61].

Conformational selection overall offers little benefit to enhance protein stability upon binding. Interestingly, this is also observed in comparing unfolding half times of corresponding unfolding simulations. The unfolding half time of bound TAD (1.539 ns) is comparable to that of apo-TAD(1.473 ns). The unfolding half time of bound NCBD is also similar to that of apo-NCBD. The transition states between bound and apo states for NCBD and TAD are similar and denature-like. The Φ-values of helices α1, α2, α3, α4, and α5 between bound and apo states have not significant different. Therefore, high-temperature unbinding kinetics data further support a global conformational selection mechanism in the NCBD-TAD interactions.

The folded state confirms the local conformational change (shown in Figure 3). The results suggest that the significant differences for NCBD and TAD are mostly focused on C terminal region. The free energy landscape for bound and apo states also confirms local conformational difference. Overall, these results support the existence of local induced fit for the recognition between NCBD and TAD.

### Conclusions

Molecular dynamics (MD) simulations are used to study the recognition mechanism between intrinsic disordered proteins. The average RMSD values between bound and corresponding apo states and Kolmogorov-Smirnov *P* test analysis indicate that TAD and NCBD may follow a global conformational selection mechanism. Quantitative analysis indicates that the magnitude of conformational selection is more pronounced than that of induced fit interaction at global level. The local magnitude of conformational selection is smaller than that of induced fit for TAD and NCBD. This suggests that both TAD and NCBD have local conformational optimization. Therefore, the recognition between TAD and NCBD might follow global conformational selection and local induced fit. These conclusions are further supported by high-temperature unbinding kinetics and room temperature landscape analysis. These methods can be used to study the recognition mechanism of other intrinsic disordered proteins.

## Supporting Information

Figure S1 A. The binding free energy for each residue. B. Relative magnitude of conformational selection and induced fit. A histogram of average RMSD value represents the magnitude of conformational selection or induced fit. The parameter △ represents the probabilistic weighting differences between conformational selection and induced fit. A: NCBD. B: TAD.
(DOCX)
