# Correction: Global Conformational Selection and Local Induced Fit for the Recognition between Intrinsic Disordered p53 and CBP

**DOI:** 10.1371/annotation/2212a861-273a-4c34-816a-ead5d0d8a7f5

**Published:** 2013-12-10

**Authors:** Qingfen Yu, Wei Ye, Wei Wang, Hai-Feng Chen

Tables 1 and 2 were erroneously omitted from the paper. The Tables are available below.

Table 1: http://plosone.org/corrections/pone.0059627.t001.cn.tiff

Table 2: http://plosone.org/corrections/pone.0059627.t002.cn.tiff

Additional text was incorrectly added to the Figure 1 Legend. The correct Figure 1 legend is:

**Figure 1. Ribbon representation of the NMR structure for TAD/NCBD complex (pdb code: 2L14).** Helices α1, α2 and α3 of NCBD are colored with blue, cyan and green, respectively. Helices α4 and α5 of TAD are colored with yellow and red, respectively. N and C-terminal domains are labeled.

