# Supplementary material for: Global Conformational Selection and Local Induced Fit for the Recognition between Intrinsic Disordered p53 and CBP
Source: PLoS One. 2013 Mar 26;8(3):e59627. doi: 10.1371/journal.pone.0059627 (PMC3608666; doi:10.1371/journal.pone.0059627)
Supplement: Figure S1 — A. The binding free energy for each residue. B. Relative magnitude of conformational selection and induced fit. A histogram of average RMSD value represents the magnitude of conformational selection or induced fit. The parameter △ represents the probabilistic weighting differences between conformational selection and induced fit. A: NCBD. B: TAD. (DOCX) [file pone.0059627.s001.docx]

Figure S1A. The binding free energy for each residue.

Figure S1B. Relative magnitude of conformational selection and induced fit. A histogram of average RMSD value represents the magnitude of conformational selection or induced fit. The parameter △ represents the probabilistic weighting differences between conformational selection and induced fit. A: NCBD. B: TAD.
